# Iron as Therapeutic Target in Human Diseases

**DOI:** 10.3390/ph12040178

**Published:** 2019-12-05

**Authors:** Raffaella Gozzelino, Maura Poli, Paolo Arosio

**Affiliations:** 1 Chronic Diseases Research Center (CEDOC)/NOVA Medical School, Universidade NOVA de Lisboa, 1180-052 Lisbon, Portugal; raffaellagozzelino@gmail.com; 2 University of Brescia, DMMT, Viale Europa 11, 25123 Brescia, Italy; maura.poli@unibs.it

Iron is essential for almost all organisms, being involved in oxygen transport, DNA synthesis, and respiration; however, it is also potentially toxic via the formation of free radicals. Thus, iron homeostasis is tightly controlled by mechanisms that have been partially elucidated. Numerous disorders have recently been linked to deregulation of iron homeostasis, leading iron metabolism to become an interesting therapeutic target for novel pharmacological treatments against these diseases. The targeting includes the hepcidin/ferroportin axis for the regulation of systemic iron homeostasis, the cytosolic machinery for the regulation of intracellular iron status and oxidative damage, proteins of iron metabolism such as ferritin and transferrin receptor, and the recently described form of programmed cell death named ferroptosis. Because of its tight link with anemia, iron metabolism has been mainly of interest in terms of the hematological pathologies, but, recently, clinicians have become aware of the importance of iron in non-hematological disorders, suggesting that iron could be a therapeutic target for various conditions. To verify whether this is the case and to try to gather together all the novel information in this developing field, we launched this issue in *Pharmaceuticals*, and we were glad to find that this attracted the attention of the 49 research groups that proposed papers. This result confirms the increasing importance of iron in various disorders.

From the articles received, it became clear that one of the fields that started to be highly investigated is the role of iron in the brain. Indeed, the recent evidence that excess iron occurs in the brains of subjects with neurodegenerative disorders suggested the therapeutic potential of iron targeting in this organ. In this issue, Crichton et al. [1] reviewed studies on iron chelators, which have been successful at removing excess iron from liver, spleen, heart, and pituitary gland in various iron-loading disorders. Oral deferiprone has been used to chelate brain iron overload in Parkinson’s disease and Friederich’s ataxia with encouraging results, and new and safer chelators are under study. The authors also suggested that the presence of inflammation might reduce the efficacy of these chelators, which is in agreement with other literature findings. Similarly, Nunez et al. [2] focused on the properties required for an optimal iron chelator to treat neurodegenerative diseases involving brain iron accumulation, a group of disorders that in addition to the already cited Parkinson’s disease and Friedreich’s ataxia, also includes pantothenate-kinase-associated neurodegeneration, Huntington’s, and Alzheimer’s disease (AD). While the ideal chelator should target the mitochondria, quench free radicals, have micromolar-iron-binding affinity, and be selective for neuronal cells, the current situation on available chelators was discussed by the authors in their manuscript. Studies on specific brain pathologies have also been included in this issue, as the work by Alsina et al. [3], which focused on the role of iron in Friedreich’s ataxia, a genetic disorder caused by trinucleotide GAA expansions in the first intron of the frataxin gene that decrease its expression. In addition to discuss the proposed functions of this mitochondrial protein and how it is related to iron homeostasis, they also reported the beneficial effects of iron chelators as therapeutic agents for this disease. The role of iron in AD was reviewed by Masaldan et al. [4], who pointed out iron dyshomeostasis as a critical feature of this disease, given the participation of iron in the generation of free radicals and in the interaction with proteins known to cause of AD pathology. The hypothesis described by the authors is that iron accumulation might derive from an age-associated increase of senescent cells that drive inflammation, which predisposes to oxidative stress, cellular dysfunction and iron-dependent death. Elevated brain iron is associated with AD progression and cognitive decline, and it may be reduced pharmacologically. Nnah et al. [5] considered that microglia activation and secretion of proinflammatory cytokines are hallmarks of neurodegenerative disorders, including AD. However, it is still unclear whether increased brain iron augments the inflammatory responses of microglia and how these cells accumulate, store, and utilize intracellular iron to carry out their functions under normal and pathological conditions. This review describes known and emerging mechanisms involved in microglial cell iron transport and metabolism, as well as the inflammatory responses affecting the brain, in the context of AD. Neurodegeneration with brain iron accumulation (NBIA) was reviewed by Levi and Tiranti [6]. NBIA is a group of rare monogenetic diseases that are heterogeneous in onset and symptoms but characterized by specific brain iron deposition in the region of the basal ganglia. Fifteen NBIA genes have been described so far, but only two of these code for iron proteins. The review reports the recent data on new models of these disorders aimed at understanding the pathogenesis of iron deposition. The impairment of mitochondrial function, which also contributes to cause neurodegeneration, is discussed in the review published by Fiorito et al. [7], who proposed a new perspective regarding the impact of heme. Heme is synthesized in the mitochondria and its metabolism plays a central role in organelle function. Since some evidence indicates that alterations of heme metabolism are associated with neurodegenerative disorders, these studies may open new therapeutic avenues in the struggle against these disorders. 

The importance of iron in the development of human brain was also described in this issue, in which Markova et al. [8] reviewed the recent findings on the effects of gestational and lactational iron deficiency on the correct formation of the central nervous system. In early embryonic life, iron is needed for the developing brain, which is characterized by a widespread expression of transferrin receptors. If iron deficiency occurs at this stage, the brain may not fully develop, weight less and present an impaired myelin formation, which result in chronic and irreversible damage affecting individual cognitive, memory, and motor skills. Studies on humans and animal models suggest the possibility to reverse these effects with iron substitution therapy. Rockfield et al. [9] argued that the maintenance of iron and lipid homeostasis is critical also to the brain. Since this is the fattiest organ in the body, many evidences point out the existence of a cross-talk between these pathways. In this article, the authors discuss human diseases involving iron and lipid alterations, with special emphasis on neurodegenerative disorders, and the therapeutic potential of iron reduction techniques for these patients. The mechanisms of iron action in the brain was also described by Ferreira et al. [10], who took a broader approach exploring in particular the cognitive and behavioral implications of disruption of iron homeostasis on the onset and progression of psychosocial disorders. In this review, the authors also discuss the links between iron and the biological, psychological, and social dimensions that contribute to the development of a diverse set of neuro-pathologies. Potential avenues of research are also outlined. Genetic diseases with brain iron accumulation were reported to also cause retinal degeneration, as discussed by Shu and Dunaief [11]. Iron dysregulation in the eye might also occur upon dietary or parenteral supplementation, which has been reported to elevate iron levels in the retinal pigment epithelium (RPE) and to promote retinal degeneration. While studies in mice and humans suggest that iron toxicity might contribute to the pathogenesis of age-related macular degeneration, iron chelators were found capable to protect photoreceptors and RPE in mouse models. So, their therapeutic potential is currently under investigation. 

Although hereditary hemochromatosis (HH) is the first iron overload disorder to be studied, there are still many aspects to be investigated, some of which considered in this issue. Loreal et al. [12] reviewed works on HH, which are mainly related to the C282Y mutation in the *HFE* gene. This mutation causes hepcidin deficiency and iron accumulation in liver, pancreas, heart, and bone. Treatment mainly consists of venesection for the removal of iron contained in red blood cells, which seems to be effective. Nevertheless, new approaches targeting hepcidin levels could be useful to better control iron parameters and especially some symptoms of this disease, like arthritis. The work by Porto et al. [13] described a 20 year follow up of three siblings, diagnosed with HH in their childhood, who were homozygous for the C282Y mutation of *HFE*. These patients were assessed yearly for the determination of iron indices and lymphocyte counts, and the analyses revealed an important transition of this disease from childhood to adult life. Emphasis on the tight link between iron status and the activation of immune cells was given by the authors. HH with dominant transmission, which is also known as ferroportin disease, was reviewed by Vlasveld et al. [14]. The authors reported phenotypes ranging from a loss of ferroportin function (LOF) to a gain of function (GOF) of this gene, with hepcidin resistance. The analyses of 359 patients with 60 ferroportin variants allowed the authors to conclude that the phenotypes of hepcidin-resistant GOF variants were indistinguishable from the other types of HH. While these can be categorized as ferroportin-associated HH, ferroportin disease may be confined to the LOF variants. Although many proteins are involved in the regulation of iron homeostasis, Katsarou and Pantoupolos [15] considered hepcidin as an interesting therapeutic target. Genetic defects of hepcidin expression lead to “hepcidinopathies”, a series of pathologies ranging from HH to iron-refractory iron deficiency anemia. Indeed, dysregulation of hepcidin is a cofactor in iron-loading anemias with ineffective erythropoiesis and anemia of inflammation. Hence, this review summarized the state of the art on hepcidin agonists and antagonists, as well as inducers and inhibitors of hepcidin expression. The interaction between hepcidin and ferroportin in the regulation of systemic iron homeostasis was also the focus of the study published by Hawula et al. [16]. Indeed, this axis can be affected by various stimuli and its deregulation can lead to a variety of disorders, which include HH. The treatment options for regulating iron levels in patients are limited, and efforts are being made to uncover approaches restoring hepcidin and ferroportin expression. In this review, the authors examined the current status of hepcidin and ferroportin agonists and antagonists, as well as inducers and inhibitors of these proteins and their regulatory pathways. Investigating the types of cellular events occurring in the liver during iron overload conditions, Tangudu et al. [17] examined the hepatic signaling pathways underlying acquired and genetic iron overload disorders. The authors found an association between these pathologies and the decline in the activation of the mitogen-activated protein kinase (MAPK)/extracellular signal-regulated kinase (Erk) kinase (Mek1/2) pathway, which selectively affects the phosphorylation of Erk1/2. The uncoupling of this signaling from iron-Bmp-Smad-mediated hepcidin induction indicates that it may contribute to a number of liver pathologies. A new approach to study the hepcidin-binding proteins was proposed by Diepeveen et al. [18], who observed that this method, in the serum, might influence hormone function and quantification. The authors used peritoneal dialysis to measure freely circulating solutes in blood and peritoneal fluid of patients undergoing a peritoneal equilibration test. The protein-bound fraction of hepcidin was calculated to be 40% (±23%), which led the authors to conclude that a substantial proportion of hepcidin is freely circulating.

Other genetic disorders associated with dysregulated iron metabolism are those characterized by new mutations in the ferritin L gene, as described by Cadenas et al. [19]. These pathologies cause dominant L-ferritin deficiencies and hereditary hyperferritinemia cataract syndrome (HHCS). An accurate diagnosis is needed for the appropriate treatment of the multiple phenotypes caused by FTL gene mutations, and a novel diagnostic algorithm was proposed in this study for that purpose. As discussed by Chiou and Connor [20], ferritin is the main iron cellular storage molecule in the body, able to store a large amount of iron within its mineral core. Recently, ferritin was shown to have a range of abilities that go well beyond iron storage. This review aims at discussing novel functions and biomedical uses of ferritin in the processes of iron delivery, delivery of biologicals such as chemotherapies and contrast agents, and the utility of ferritin as a biomarker in a number of neurological diseases. The autophagic degradation of ferritin (known as “ferritinophagy”) is necessary to maintain intracellular iron homeostasis and is mediated by the nuclear receptor coactivator 4 (NCOA4), as described by Santana-Codina and Mancias [21]. In this review, the authors related the biochemical regulation of NCOA4, its contribution to physiological processes, and its role in disease. Its potential to activate or inhibit ferritinophagy and ferroptosis for therapeutic purposes was also addressed. In line, Bou-Abdallah et al. [22] reviewed our current understanding of iron mobilization from ferritin by various reducing agents. The authors reported recent results supporting a mechanism that involves a one-electron transfer through the protein shell to the iron mineral core. The physiological significance of the iron-reductive mobilization from ferritin by the non-enzymatic FMN/NAD(P)H system is also discussed. Sha et al. [23] studied plant ferritin, a novel form of iron supplement, the absorption of which can be affected by phenolic acids of the plant. The authors found that cinnamic acid derivatives induce the release of iron from soybean ferritin, thus having a negative effect on iron stability. The authors also pointed out that the iron chelating activity and reducibility of these compounds may affect the iron availability of soybeans. Regulation of iron homeostasis is also provided by the role exerted by the transferrin receptor 2 (Tfr2), as described by Roetto et al. [24]. Tfr2 is one of the hepcidin regulators and mutations of this gene cause type 3 HH. While this review summarized the data on Tfr2 extrahepatic role, including the importance of the two main isoforms, Tfr2α and Tfr2β, the use of Tfr2 as therapeutic target for hepcidin control is also discussed. Finally, the systemic iron regulation, achieved by controlling heme metabolism, was described by Montecinos et al. [25], who reviewed the present knowledge on the heme-binding protein hemopexin (HPX) and provided information on its biochemistry. HPX prevents the toxicity induced by hemoglobin (Hb)-derived heme, which occurs in hemolytic conditions and can be triggered by the activation of the immune system. The review highlights some newly identified actions of heme and HPX, engaged especially when normal processes fail to maintain heme and iron homeostasis. The authors also presented data showing that the cytokine IL-6 cross talks with activation of the c-Jun N-terminal kinase pathway in response to heme–hemopexin in models of hepatocytes.

Recent studies have shown the importance of iron in kidney diseases. This particular topic was discussed by Vera-Aviles et al. [26], who reviewed evidence for iron-induced toxicity in chronic kidney disease (CKD) and the mechanisms by which histidine exerts cytoprotective functions. In fact, CKD is often associated with iron and histidine deficiency. This amino acid, which is essential for erythropoiesis and to enhance iron dietary absorption, was shown to have antioxidant properties capable to improve the oxidative stress in CKD. Balla et al. [27] discussed some of the most important findings relating to the role of iron and ferritin heavy chain in the context of kidney-related diseases and, in particular, in vascular calcification. The authors provided evidence that the ferroxidase activity of ferritin prevents this frequent complication of CKD. Nuhu and Bhandari [28] reviewed a specific cardio-renal morbidity in CKD, providing an understanding of the pathophysiology and impact of uremic toxins, inflammation and anemia, on oxidative stress. Anemia in CKD increases the risk of left ventricular hypertrophy and oxidative stress, thereby magnifying the deleterious consequences of uremic cardiomyopathy. This enhances its progression and increases the risk of sudden cardiac death.

Iron toxicity also affect the lungs. The continuous exposure of this organ to oxygen turns it highly sensitive to oxidative damage, which is enhanced in the presence of excess iron. This was discussed by Zhang et al. [29], who provided an overview of systemic and local lung iron regulation. The authors described the role of this metal in the development of lung infections, airway disease, and lung injury, offering important foundations for the development of therapeutic applications. Also Neves et al. [30] reviewed the current knowledge on the regulation of pulmonary iron homeostasis. In addition to report the functional importance of iron and its link in the development of lung disorders, the authors provide a better understanding of the association between pulmonary iron deregulations and the frequently correlated chronic obstructive pulmonary disease and lung cancer. Possible improvement of these pathologies with iron-related therapeutic strategies has also been described.

The interplay between iron and inflammation, elicited in the course of the infection, has long been observed. Petzer et al. [31] reviewed the association between anemia and chronic inflammatory diseases, addressing how iron levels could be improved with the resolution of the disease, supplementation and redistribution strategies. Moreover, investigations referring to the key role of hepcidin in these forms of anemia encouraged the development of novel therapeutic approaches, which were discussed in this review along with the current guidelines of iron replacement therapies. In chronic inflammatory diseases, these refer to oral versus parenteral iron supplementation. The authors also reported the emerging potential of hepcidin-antagonizing drugs, which are currently under preclinical and clinical investigations. Hepcidin regulation has been also reported as beneficial in patients with rheumatoid arthritis (RA), who are often treated with an anti-IL-6-receptor (anti-IL-6R) monoclonal antibody (tocilizumab) that inevitably influences iron metabolism. Ribeiro et al. [32] studied a cohort of patients under this treatment, identifying the association between higher serum iron and transferrin saturation, induced by the drug, and the risk of infection. Their study strongly indicates the need to monitor iron indices in RA patients on anti-IL-6R therapy to prevent infections. The most recent literature on infection and iron metabolism has been reviewed by Gomes et al. [33]. Special emphasis was given to iron changes induced by pathogens invasion, which culminate with the development of anemia, and to potential therapeutic approaches that, modulating iron metabolism, correct iron levels and control the infection. One of the most studied infectious diseases involving disruption of iron homeostasis is malaria. The increase in hepcidin during the development of this diseases has been reported by Muriuki and Atkinson [34], along with the higher levels of tumor necrosis factor-α, as the cause that leads to poor iron absorption and recycling. While this may be an important driving factor of iron deficiency, its prevalence in children increases over a malaria season and decreases when it is interrupted, as described by the authors. Once the link between malaria and iron deficiency will be formally demonstrated, it would aid readjusting priorities for programs towards prevention and treatment of iron deficiency, which will benefit malaria control. Armitage and Moretti [35] reviewed the demand and supply of iron during early childhood, addressing its importance in aspects that refer to the physiology and development of young children coming from low- and middle-income countries, in particular. Thus, discussing the implications for interventions to improve iron status whilst minimizing infection-related risks is of utmost importance, since strategies should be adapted according to iron deficiency, inflammation status, and infection risk. It is known that macrophages play a central role in regulating iron homeostasis, especially during infections, like but not restricted to malaria. In their manuscript, Recalcati et al. [36] described how macrophages control iron levels and how this determines, in turn, their plasticity. Changes in the expression of genes coding for major proteins of iron metabolism may result in different iron content availabilities for the macrophage itself and other cells present in the microenvironment. This review also discussed the role of macrophages in immunometabolism, which cross-talks with erythropoiesis as reported by Sukhbaatar and Weichhart [37]. Coordination between spleen, liver, and bone marrow is essential for macrophages ensure proper iron recycling and erythroblast differentiation. This article also focuses on the role of distinct macrophage populations to maintain iron metabolism, describing the cellular and systemic mechanisms involved in iron-regulating processes. Macrophages are also the main target of *Mycobacteria Tuberculosis*, a pathogen that requires iron to proliferate, as pointed out by Agoro and Mura [38], who reasserted that *Mycobacteria*-infected hosts use systemic iron restriction and cellular iron distribution as defense mechanism against infection. The authors reviewed the importance of iron availability to elicit an immune response against *Mycobacteria*, which then dictates host susceptibility. Hence, the need for future therapeutic directions capable to prevent this disease were also pointed out. At this regards, specific chelators against infections caused by *Mycobacterium avium* have been reviewed by Rangel et al. [39], who described in particular a selected class of the 3-hydroxy-4-pyridinone ligand, which could be functionalized with the addition of fluorophores. This was shown to improve antimycobacterial activity and the affinity of chelators to biological membranes, thus indicating that “to label means to change”. The authors further discuss the need of combined therapeutic approaches and the use of rhodamine B conjugates to target bacterial resistance and biofilm production.

More evidences point at iron deficiency as critically involved in the pathogenesis of different conditions. An example is provided by Lakhal–Littelton [40], who described the prevalence of iron deficiency in patients with cardiovascular disease and associated it with worse outcomes. Although, the mechanisms by which iron deficiency affects cardiovascular function are still unclear, this review discusses the benefits of therapeutic strategies aimed at restoring cellular iron homeostasis rather than approaches based on iron supplementation. These have been described in particular on two diseases: chronic heart failure and pulmonary arterial hypertension. Another compartment also affected by iron deficiency is the bone. As explained by Balogh et al. [41] bone homeostasis is based on the regulation between osteoclasts’ function, which resorb the bone, and osteoblasts’, which produce new bone. Both iron deficiency and iron overload disrupt this delicate balance, influencing skeletal health and emphasizing the need to develop novel therapeutic approaches to inhibit the pathological effects of altered iron levels in this tissue.

An important aspect of restoring iron homeostasis is how to supplement this metal in case of deficiency, since possible side effects of oxidative damage and changes in intestinal microbiota have been pointed out. The effects of oral treatments on iron deficiency were reviewed by Ginanjar et al. [42], who also considered the potential toxicity of plasma non-transferrin-bound iron (NTBI). The authors found that FeSO4 is more absorbed than NaFeEDTA, although causes a remarkable increase of NTBI. In a double-blind, randomized trial, they showed that a low dose of NaFeEDTA (6.5 mg), given with a meal, was highly effective for the treatment of iron deficiency, maintaining normal levels of NTBI. Bhandari et al. [43] discussed the inefficacy of oral iron replacement therapies in the treatment of some patients with iron deficiency. In these cases, replacement with intravenous (IV) iron therapies, now in their third generation, could increase iron levels without causing toxic effects. This review described the properties of different IV irons, and how differences in formulations might impact the current and future clinical practice. Novel innovative oral iron formulations were described by Gomez-Ramirez et al. [44]. Sucrosomial® iron (SI), in which ferric pyrophosphate is protected by a phospholipid bilayer plus a sucrester matrix (sucrosome) and absorbed via para-cellular and trans-cellular routes (M cells), was shown to increase iron bioavailability while having excellent gastrointestinal tolerance. An important concern, though, needs to be raised from a human nutritional point of view. The genetic selection for large litter sizes and high birth weights makes piglets severely iron-deficient, as described by Szudzik et al. [45]. In need for iron supplementation, these animals receive intramuscular injection of a large amount of iron dextran, which if from one side corrects the iron deficiency of the animal, on the other it may generate toxic effects. Whether this might also affect, in long term, the human population eating pork meat is not known. Therefore, new iron supplements need to be considered, turning iron-deficient piglets as a convenient animal model for pre-clinical studies. The influence of food compounds on iron absorption was also the focus of Lesjak and Srai’s review [46], which discussed how iron homeostasis is affected by several dietary factors, such as flavonoids. Their ability to modulate the expression and activity of proteins involved in the systemic regulation of iron metabolism and uptake turn flavonoids clinically relevant for the potential treatment of both anemia and iron overload diseases. The influence of dietary iron absorption in the gut was discussed by Yilmaz and Li [47], who described the dynamic modulation of intestinal microbiota induced by different iron levels. The authors reviewed the current understanding of the effects of luminal iron on host–microbe interactions in human health and disease. The side effects induced by the excessive amount of unabsorbed iron at the interactive host–microbe interface of the human gastrointestinal tract was particularly described. 

The involvement of iron in the development of tumors has long been studied, and Busti et al. [48] consider that anemia in cancer is multifactorial, and iron deficiency (ID) is a major contributor. Since the treatment of functional iron deficiency is complex and still controversial, this work discusses the possible approaches for the management of ID in cancer patients, in different clinical settings. Current guidelines and recommendations were also reported to emphasize the need for further research in the field. In agreement, one article in this issue studied the activity of the anticancer drug didox, which is thought to act by inhibiting ribonucleotide reductase, the rate-limiting enzyme for dNTP synthesis that is highly expressed in aggressive tumor cells. Asperti et al. [49] showed that didox cell killing was suppressed by iron supplementation, and capable to reduce iron availability by acting as an iron chelator. The authors indicated that this property might contribute to its antitumor activity by sequestering iron to enzymes, as the ribonucleotide reductase.

Altogether, this issue, which was published in *Pharmaceuticals*, provides an interesting overview on the complexity of the role of iron in health and disease conditions, emphasizing the need to control iron homeostasis. This is achieved by supplying iron in sufficient amount, when deficient, and removing it, when in excess. Regulating iron distribution among various tissues and compartment is also essential to prevent dysregulated levels of this metal and the occurrence of disorders like HH, neurodegenerative and cardiovascular diseases, cancer, and infections.
